# The individual and contextual determinants of the use of telemedicine: A descriptive study of the perceptions of Senegal's physicians and telemedicine projects managers

**DOI:** 10.1371/journal.pone.0181070

**Published:** 2017-07-21

**Authors:** Birama Apho Ly, Ronald Labonté, Ivy Lynn Bourgeault, Mbayang Ndiaye Niang

**Affiliations:** 1 Population Health Program, University of Ottawa, Ontario, Canada; 2 Public Health Teaching and Research Department, University of Sciences, Techniques and Technologies of Bamako, Bamako, Mali; 3 Faculty of Medicine, University of Ottawa, Ontario, Canada; 4 Telfer School of Management, University of Ottawa, Ontario, Canada; 5 Telemedicine Research and Expertise Interdisciplinary Centre, Faculty of Medicine, Cheikh Anta Diop University, Dakar, Senegal; University of Groningen, University Medical Center Groningen, NETHERLANDS

## Abstract

Telemedicine is considered to be an effective strategy to aid in the recruitment and retention of physicians in underserved areas and, in doing so, improve access to healthcare. Telemedicine’s use, however, depends on individual and contextual factors. Using a mixed methods design, we studied these factors in Senegal based on a micro, meso and macro framework. A quantitative questionnaire administered to 165 physicians working in public hospitals and 151 physicians working in district health centres was used to identify individual (micro) factors. This was augmented with qualitative descriptive data involving individual interviews with 30 physicians working in public hospitals, 36 physicians working in district health centres and 10 telemedicine project managers to identify contextual (meso and macro) factors. Physicians were selected using purposeful random sampling; managers through snowball sampling. Quantitative data were analyzed descriptively using SPSS 23 and qualitative data thematically using NVivo 10. At the micro level, we found that 72.1% of the physicians working in public hospitals and 82.1% of the physicians working in district health centres were likely to use telemedicine in their professional activities. At the meso level, we identified several technical, organizational and ethical factors, while at the macro level the study revealed a number of financial, political, legal, socioeconomic and cultural factors. We conclude that better awareness of the interplay between factors can assist health authorities to develop telemedicine in ways that will attract use by physicians, thus improving physicians’ recruitment and retention in underserved areas.

## Introduction

As with many countries, Senegal suffers from an inadequate supply and misdistribution of physicians [1,2]. Eighty percent of its specialist physicians work in Dakar, the national capital, yet this region counts for only 23% of its population [2]. Certain specialist physicians are available only in Dakar [2]. To access these physicians, patients from underserved areas sometimes have to travel very long distances. This situation can negatively affect their health and exacerbate health inequities [3]. Prior research on this topic suggests that the misdistribution of physicians in Senegal is influenced by a combination of professional, personal, family and community factors [4]. Professional factors include isolation, disagreement with where they are assigned to practice, extended stay in underserved areas, and lack of opportunities for professional development. Personal factors include the individual reasons why physicians choose to not work and not stay in underserved areas. Family factors comprise issues such as the lack of good schools for children and jobs for spouses, while community factors include difficulties physicians may experience in integrating within local communities. Uneven distribution of physicians in Senegal is further affected by a high rate of emigration, with more than the half of Senegalese trained physicians estimated to be working abroad [5].

In recent decades, several measures were adopted in Senegal to increase the number of physicians in underserved areas, which aimed essentially at improving the production and management of physicians [4]. To date, however, none of these measures have given satisfactory results with a disproportionate number of physicians still located in Dakar. This situation compels decision-makers, planners, and researchers to find other solutions to improve the distribution of physicians between the underserved and other areas.

Several researchers over the past decade have been studying telemedicine and consider it to be a potential solution to improving the recruitment and retention of physicians in rural and historically underserved regions [6,7]. The technology can reduce their professional isolation, allow them to get experts’ opinions from a distance, and decrease their workload [8–11]. By aiding recruitment and retention, telemedicine can also improve equitable access to healthcare [12,13].

Telemedicine considerations have been present in Senegal for some time, with several projects attempted over the past two decades. Most of these projects, however, ended in their early stages with the use of telemedicine now limited to just a few public hospitals and district health centres. The reasons why the technology has not spread more widely in the health system are not well known. Many factors have emerged from previous research including individual, technical, organisational, financial, ethical, legal, political and socio-cultural influences [14–16]. Broens et al. describe individual factors as those that influence the decision to use telemedicine; technical factors as those related to the support, training, usability of equipment and the quality of the technology used in telemedicine; financial factors as investment, maintenance and operating costs of telemedicine; and organizational factors as internal and external changes following the introduction of telemedicine [14]. Broens et al. describe political and legal factors as the laws, policies, and standards related to the use of telemedicine. This typology provides a useful framework for analyzing the existing state of knowledge about telemedicine use (not restricted to Senegal), and for discussing the results of our own study [14].

## Objectives

This study pursued two objectives. The first objective focused on the individual factors and the second on the contextual factors that determine the use of telemedicine in Senegal. Individual factors referred to the intention of Senegal’s physicians to use telemedicine in their professional activities. Contextual factors referred to the technical, organizational, ethical, financial, political, legal and socioeconomic factors that influence the use of telemedicine in Senegal.

The main goal of this study was to gain greater insight on the determinants of telemedicine use in Senegal to better inform decision makers, researchers, telemedicine projects managers and health professionals on the factors on which actions could be undertaken to better encourage the use of telemedicine in the country. As noted earlier, the assumption is that improving the use of telemedicine will likely enhance the recruitment and retention of physicians in underserved areas, improving access to healthcare for the rural population and, in turn, overall population health.

## State of knowledge

Utilizing the categories from Broens et al. on the factors influencing telemedicine use, we summarize the current literature. What becomes clear from this review is that most of these studies took place in high-income countries, and only a few examined these factors in countries with similar resource-constraints such as those experienced in Senegal.

### Individual influences

Health professionals can have a negative attitude regarding telemedicine by doubting its effectiveness, fearing their patients’ safety and privacy, preferring traditional methods of care, or doubting the ability of telemedicine to ensure their wages. They might also perceive telemedicine as taking more time and increasing their workload [17]. Patients can also have a negative attitude regarding telemedicine, resulting in their refusal to participate in telemedicine projects and affecting physician attitudes towards the technology.

### Technical factors

There are a number of technical factors that affect the use of telemedicine. One of the best-known factors is the quality of internet connection [6,18]. Another technical factor is the lack of training [19]. This factor is seen as one of the most important barriers to telemedicine use because, in general, health professionals are not trained in use of this technology during medical school [6]. A third technical factor is the frequency of technical failures. In a study published in 2001, technical failures were reported in 17% of telemedicine consultations [20]. These technical failures may cause delays in the use of telemedicine [18], or discourage some telemedicine services users [6].

### Organisational influences

There are many organizational factors that influence the use of telemedicine. One of the best-known barriers is the lack of time [21–23], with other factors including work overload, again widely recognized as one of the most important barriers to the use of telemedicine [24,25]. There are also the organizational changes following the introduction of telemedicine in healthcare systems, which can upset habits, impose internal and external changes in services organization and negatively influence telemedicine use [14,26].

### Financial considerations

Some of the main financial factors are the investment, operational and maintenance costs related to telemedicine [14]. These costs can be very high [27], which may limit their full implementation. There is also the issue of compensation for telemedicine providers, with many governments refusing to reimburse physicians practicing telemedicine [28], thereby discouraging. This situation may discourage some physicians from using it [29]. There is equally the lack of stable funding. Many telemedicine projects are financed by governments, universities, hospitals, telecommunication operators, equipment manufacturers, international and regional organizations, semi-commercial organizations and armies [30]. The funding is often short-term and not sustainable, which leads to the early failure of many projects [14,19].

### Ethical concerns

The best-known ethical factor concerns data security, which is often cited as one of the main causes of physicians’ resistance towards telemedicine [31]. Another factor is the lack of an ethical framework to guide the use of telemedicine [32].

### Political concerns

The political factors that influence the use of telemedicine are the collaboration and commitment of political actors [33], government policy [34], legislation [14] and standardization [14]. Standardization ensures quality and uniform practice, but standards are not always available or enforced [14].

### Legal influences

Concerns over the liability of telemedicine providers has been mentioned by many authors and extends to civil, penal, disciplinary and ordinal liability [35]. There is also the lack of a legal framework for telemedicine care in many countries [32] which may discourage the use of telemedicine or fail to prevent harmful practices in its use.

### Socioeconomic factors

Finally, there is a broader range of socioeconomic factors which may affect telemedicine use, including cultural and religious beliefs Rural patients generally prefer practitioners who are familiar with their culture [36]. Socioeconomic factors also include poverty since people with low income will not be able to pay telemedicine costs if they are high and not available as a free service within a public program [30,36]. Health worker strikes and social conflicts can also disrupt telemedicine use. These problems are known for their ability to paralyze entire health systems [37–39].

## Conceptual framework

**Fig 1:**
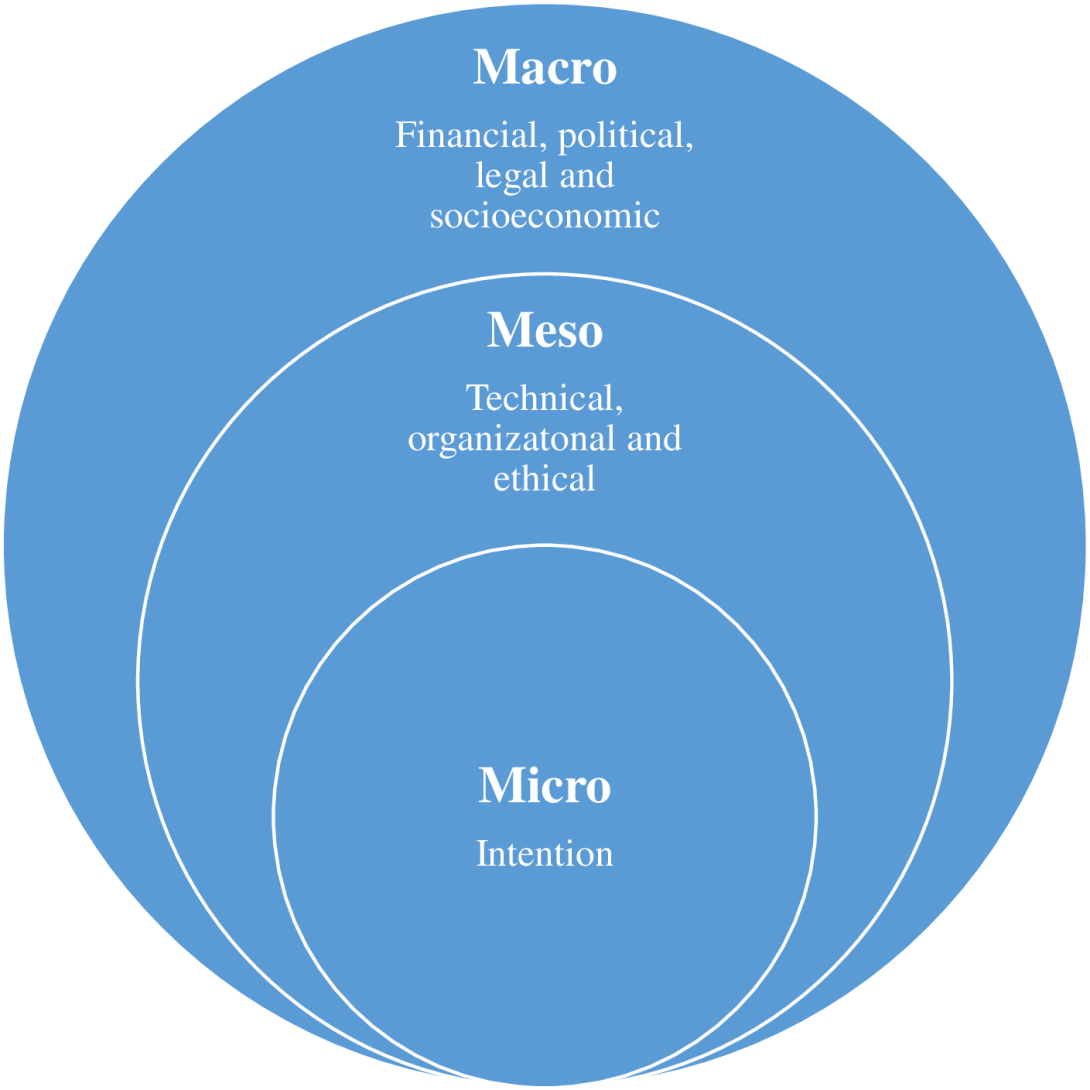
The micro, meso and macro framework of the use of telemedicine. The use of telemedicine is influenced by micro, meso and macro levels factors.

We framed our analysis of factors influencing telemedicine use using a micro, meso and macro socioecological framework (Fig 1). This framework is an adaptation of the Dahlgren & Whitehead model, which is one of the most widely known and used frameworks in population health [40]. It organizes the various factors that influence individuals’ health (and behaviour) into five categories and presents them in a series of five levels structurally one on top of the other, but nevertheless intersecting. The upper level includes the major structural factors such as the general socioeconomic, cultural and environmental conditions. The level below includes the material and social conditions in which people live and work such as education, housing, and healthcare. The third level includes social and community support such as mutual support from family, friends, neighbours and the local community. The next level corresponds to individuals’ behaviour such as their eating, smoking and drinking habits while the lowest level represents individuals’ characteristics such as age, sex, genetic make-up which are regarded as fixed, and so outside of the ability of policy makers to control them [41]. Interventions, instead, tend to focus on the upper four levels.

The micro, meso and macro layered conceptual framework for this research follows the same logic. It proposes three categories of factors and layers them in three levels, one on the top of the other. In this study, the macro-level includes the financial, legal, political and socio-cultural factors that influence the use of telemedicine in Senegal, which correspond to the structural factors described by Dahlgren and Whitehead in their top layer [41]. The meso-level includes the technical, organizational and ethical factors. The micro-level refers to individual factors, particularly individuals’ intention to use telemedicine. This framework is useful in that it affords insight into the various factors that influence the use of telemedicine. Also, it suggests that there is interaction between, and interdependence of, these different factors. It also proposes different points of intervention for decision makers and planners when developing telemedicine initiatives.

## Methods

### Study design

Our study design involved mixed methods, combining a quantitative questionnaire and a qualitative study involving individual interviews. Research participants comprised physicians and telemedicine project managers. In Senegal’s public health sector, the vast majority of physicians work either in public hospitals or district health centres. The participating physicians were selected using random and purposive sampling [42]. A total of 165 physicians working in public hospitals and 151 in district health centres were randomly selected to study the micro factors (quantitative questionnaire), while 30 physicians working in public hospitals and 36 in district health centres were randomly chosen to study the meso and macro factors (qualitative study). Telemedicine managers (N = 10) were purposively selected using a snowball sampling strategy for the qualitative study. The random sampling used to select physicians in public hospitals and district health centres was the systematic random sampling which consist of randomly select the first participant and systematically the other participants based on a scale.

The questionnaire employed solicited physicians’ socio-demographic and professional characteristics and asked a single question about their intention (likelihood) to use telemedicine in their professional activities, measured by their response to a seven-point Likert scale (from highly unlikely to highly likely). It (questionnaire) was administered between January and March, 2015. The semi-structured interview guide for the qualitative study comprised seven open-ended questions on the meso (technical, organizational and ethical) and macro (financial, political, legal and socioeconomic) factors that influence the use of telemedicine in Senegal. These tools were tested by the lead researcher for ambiguous and troublesome wording and assessed by an expert committee. Afterward, they were tested with a small sample of three physicians working in public hospitals, three physicians working in district health centres and three telemedicine managers in order to detect any problem related to the questions and their administration. Interviewees were allowed to talk at length, on their terms, and with enough time to reflect. Clarifications were sought whenever needed. Interviews lasted 30 to 85 minutes. All interviews were audio-recorded. Data were collected, between February and June 2014, in physicians’ office, home, hotel room, and in the restaurants, the airport and training centres.

### Data analysis

For micro factors, we performed analyses using SPSS version 23. We first conducted descriptive analyses of physicians’ characteristics and intention. During these analyses, we calculated the frequency of each characteristic of the participating physicians and estimated the mean scores by characteristic (sex, age, region of practice and specialization). We then compared these mean scores using a one-way ANOVA. For meso and macro factors, we conducted a descriptive thematic analysis on the transcripts of the interviews using NVivo 10 software. Transcripts were analyzed by the lead author for both deductive, a priori codes and inductive, emergent codes. A priori codes based on the conceptual framework included the technical, organizational, financial, ethical, legal, political and socio-economic factors. Emergent codes are described explicitly in each of the sections below.

## Results

### Participant characteristics

Tables 1 and 2 provide demographic data on the participants in the questionnaire and key informant studies, respectively.

**Table 1:** Socio-demographic and professional characteristics of the participants involved in the quantitative study (micro-factors).

Characteristics		Physicians working in public hospitals		Physicians working in district health centres	
		N	%	N	%
**Sex**	Male	112	67.88	97	64.24
	Female	53	32.12	54	35.76
**Age**	≤ 30	10	6.10	16	10.59
	31–35	39	23.64	38	25.16
	36–40	46	27.88	45	29.80
	41–45	26	15.76	20	13.24
	46–50	22	13.33	18	11.92
	> 50	22	13.33	14	9.27
**Specialization**	General practitioners	30	18.18	64	42.38
	Specialist physicians	135	81.82	74	49.01
	Trainees physicians	0	0.00	13	8.61
**Medical regions**	Dakar	125	75.76	100	66.23
	Out of Dakar	40	24.24	51	33.77

**Table 2:** Socio-demographic and professional characteristics of the participants involved in the qualitative study (meso and macro-factors).

Characteristics		Physicians/ public hospitals		Physicians/ district health centres		Telemedicine project managers	
		N	%	N	%	N	%
**Sex**	Male	24	80.00	34	94.44	10	100.00
	Female	6	20.00	2	5.56	0	0.00
**Age**	≤ 30	2	6.67	0	0	1	10.00
	31–35	3	10.00	10	27.77	1	10.00
	36–40	4	13.33	13	36.11	0	0.00
	41–45	7	23.33	7	19.44	0	0.00
	46–50	6	20.00	5	16.66	2	20.00
	51–55	7	23.33	1	0.00	2	20.00
	56–60	0	0.00	0	0.00	3	30.00
	> 60	1	3.33	0	0.00	1	10.00
**Specialization**	General practitioner	0	0.00	13	36.11	0	0.00
	Specialist physician	30	100	23	63.89	7	70.00
	Not a physician	0	0.00	0	0.00	3	30.00
**Medical Region**	Dakar	24	80.00	2	5.56	8	80.00
	Outside Dakar	6	20.00	34	94.44	2	20.00

Physicians in the study involving questionnaire were predominately men in both of the two institutional settings studies. The average age of those working in district health centres was 39 years while the average age of their colleagues who worked in public hospitals was 41 years. In the both groups, specialist physicians were more numerous than general practitioners and trainees physicians. Similarly, physicians working in Dakar were more numerous than those working outside Dakar.

Participants in the qualitative study (key informant interviews) were, again, predominately men and specialist physicians. Physicians working in district health centres were younger (40 years) than those working in public hospitals (44 years), or those who working as telemedicine projects managers (50 years). Seven telemedicine project managers were specialist physicians; three were not physicians, but were specialized in education, Information and Communication Technologies (ICT) and health business development. The physicians working in public hospitals and telemedicine project managers were mostly working in Dakar, while the physicians working in district health centres were mostly working outside Dakar.

### Micro factors

Results suggest that the majority (72.12%) of the physicians working in public hospitals were likely to use telemedicine in their professional activities and that their intention did not differ by age (F: 0.92; p: 0.59), sex (F: 1.22; p: 0.27) and specialization (F: 0.29; p: 0.75), but by region of practice (F: 3.76; p: 0.05). Table 3 shows the distribution of their intention according to their region of practice.

**Table 3:** Intention of the physicians working in public hospitals according to their region of practice.

Intention	Dakar	Outside Dakar	Total
Highly unlikely	16 (12.80%)	2 (5.00%)	18 (10.91%)
Quite unlikely	7 (5.60%)	3 (7.50%)	10 (6.06%)
Slightly unlikely	2 (1.60%)	1 (2.50%)	3 (1.82%)
Neither unlikely nor likely	14 (11.20%)	1 (2.50%)	15 (9.09%)
Slightly likely	25 (20.00%)	8 (20.00%)	33 (20.00%)
Quite likely	40 (32.00%)	10 (25.00%)	50 (30.30%)
Highly likely	21 (16.80%)	15 (37.50%)	36 (21.82%)
**Total**	**125** (100.00%)	**40** (100.00%)	**165** (100.00%)

Results from physicians working in district health centres indicate that 10 (6.6%) of them were highly unlikely, 10 (6.6%) quite unlikely, 5 (3.3%) slightly unlikely, 2 (1.3%) neither unlikely nor likely, 50 (33.1%) slightly likely, 38 (25.2%) quite likely and 36 (23.8%) highly likely to use telemedicine. These results demonstrate that 82.1% of the physicians working in district health centres were likely to use telemedicine in their professional activities, even their intention did not differ by age (F: 1.20; p: 0.25), sex (F: 0.35; p: 0.56), specialization (F: 1.16; p: 0.32) and region of practice (F: 1.10; p: 0.30).

### Meso-level factors

#### Technical factors

This study identified five technical factors that can influence the use of telemedicine in Senegal (Table 4). Unsurprisingly, telemedicine use can be prevented by a lack of computer equipment (computers, cameras, scanners, and printers), medical equipment (surgical material, etc.) or medical exploration equipment (ultrasound, X-ray, electrocardiogram, etc.). The availability of this equipment was considered by almost all of our participants as essential prerequisites to their use of telemedicine:

"If we ask for a colleague’s support for a disease without having the needed medical equipment to manage that disease, this can lead to a problem. Even if we have the diagnosis, if we don’t have the needed instruments and tools, we cannot treat the patient. Even if the colleague tells us to do this, to do that, we cannot solve the problem, unfortunately. This is important."

(Male general practitioners working in a district health centre outside Dakar)

**Table 4:** Meso- and macro-level factors identified by participants.

Level	Factors identified by key informant
**Meso**	
Technical	Lack of telemedicine equipment Lack of equipment maintenance Lack or poor quality of internet connection Lack or poor quality of electricity supply Lack of training
Organizational	Lack of information on telemedicine Undersupply of human resources
Ethical	Lack of an ethical framework for telemedicine
**Macro**	
Legal	Lack of a legal framework for telemedicine
Political	Dysfunction of the Telemedicine National Steering Committee Non-translation of political will into concrete actions Lack of consideration of telemedicine as a political priority Lack of a national telemedicine strategy
Financial	High investment, operating, training and maintenance costs Scarcity of funding sources No physician compensation for telemedicine use
Socioeconomic and Cultural	Religious and socio-cultural beliefs about telemedicine Healthcare worker strikes Social conflicts (Casamance) Patient poverty

Participants also noted that the lack of equipment maintenance was another weak link in the health system which could have a negative impact on the sustainable development of telemedicine:

"The weak point of the system is, particularly, equipment maintenance. That is at all levels. The problem of equipment maintenance is a serious problem for our health system."

(Male specialist physician working in a district health centre outside Dakar)

Internet connection was also not available in some health facilities and, when it was, its quality was poor. One of the physicians working in public hospitals said:

"Currently, I have a computer, but internet connection is interrupted. I don’t know since when. In any case, I wanted to connect, but I have been told that internet connection didn't work for one week."

(Male specialist physician working in a public hospital outside Dakar)

These participants believed that the lack of internet connection could prevent the large scale use of telemedicine and that the poor quality of internet connection could jeopardize the security of information using that technology.

Our results also found that some health facilities were not supplied with the electricity produced by the national electricity company (SENELEC). Some of these facilities worked with generators or solar panels. Others didn’t have any source of electricity. For our participants, this situation is not suited to their use of telemedicine because everything, including internet connection, depends on electricity.

"We have electricity, but sometime, there are power cuts. For example, last week, we spent three days without any electricity from 8 am to 5 pm."

(Male specialist physician working in a district health centre outside Dakar)

In health facilities where the electricity was provided by the national electricity company (SENELEC), power cuts happened frequently. For our participants, these interruptions posed a real danger for their patients.

Finally, the lack of training on the technical aspects of telemedicine, which is not always taught in medical schools, could also have a negative impact on its use:

"Telemedicine uses a particular technology. This technology is not mastered by practitioners. We are not technicians, and if we don’t master all the technology installed for telemedicine that is a problem. Therefore, it is necessary that providers are trained in the use of the technology. If not, we are heading towards trouble."

(Male specialist physician working in a district health centre outside Dakar)

#### Organizational influences

Two organizational factors emerged that can influence the use of telemedicine: the lack of information on telemedicine and an undersupply of human resources. Our participants generally reported that they were not well informed about the telemedicine interventions carried out by Senegalese health authorities. They stated that they did not have enough information on telemedicine interventions even though they believe that they are the first to be informed.

"Telemedicine is too centralized. There is no decentralization. I am in my region, almost for five years, but we never talked about telemedicine in this region. … That is a problem. There is asymmetry of information. Only the people of central level have information. At peripheral level, we don't have anything. That is a problem. We have to have certain information."

(Male specialist physician working in a district health centre outside Dakar)

For the physicians involved in this study, an undersupply of physicians and technicians in the country led to work overload, which left little or no room for the use of telemedicine.

"I used to say that the system is itself faulty since only one person is called to do several tasks at the same time. Here, I come back to the lack of human resources that compel us to be multifunctional. That is an important barrier."

(Male specialist physician working in a district health centre outside Dakar)

We are left, then, with a conundrum: Telemedicine aims to address the lack of human resources, but it needs adequate human resources itself to run effectively.

#### Ethical concerns

This study identified one main ethical factor: the lack of an ethical framework to guide telemedicine use. Our participants reported that, in Senegal at present, *there* is no ethical text governing, for example, confidentiality, medical secrets and informed consent, raising the concern:

"I really don’t know. I speak like a neophyte [but] will confidentiality be respected?"

(Female specialist physician working in a public hospital in Dakar)

### Macro factors

At the macro-level, we consider the most distal factors influencing the use of telemedicine categorised here as legal, political, financial and socioeconomic factors. These factors seem to have a direct impact on the meso-level factors, but they also seem to have an intersecting influence on each other.

#### Legal factors

According to our participants, there was no legal framework governing the use of telemedicine in Senegal, and so no regulatory text that guarantees the protection of data, patients, and providers. One of the physicians working in district health centres said:

"Are we protected with respect to that? Is the provider covered? Does the medical order cover us with respect to that? Does Health Ministry, in the case of a problem, endorse that? We don’t know what can happen to us by using it [telemedicine]. Is there any law to protect us? Do laws exist? If they exist, what is their content?"

(Male specialist physician working in a district health centre outside Dakar)

This situation, according to our participants, could create distrust toward telemedicine and eventually be a major obstacle to its use.

#### Political influences

This study describes four political factors that can influence the use of telemedicine in Senegal: the dysfunction of the Telemedicine National Steering Committee, the non-translation of political will into concrete actions, the lack of consideration of telemedicine as a political priority and the lack of a national telemedicine strategy.

Participants pointed to the challenges resulting from what they viewed to be dysfunctionality at the level of the National Telemedicine Steering Committee. Some of this was represented by the difficulties this Committee experienced in even gathering its members for meetings. Although it could be a mechanism to coordinate telemedicine efforts, the Committee has not achieved its full potential in effectively organizing and coordinating telemedicine activities at the national level. The result, according to three telemedicine projects managers, is a proliferation of telemedicine projects, which sometimes leads to unnecessary duplication of efforts.

"There is much duplication. If you go into certain health facilities, you will find three telemedicine programs."

(Male telemedicine projects manager working in Dakar)

This lack of coordination was noted by respondents across the different public hospitals and district health centres.

Our participants further believed that political will was imperceptible, timid or not translated into concrete action, creating an obstacle to the development of telemedicine.

"Yes, I think there is political will, but it remains the problem of realization. It is clear that when we contact them [politicians], they will tell us that they are in agreement. They are always in agreement, but when the time of realization comes. That is something else."

(Male specialist physician working in a public hospital in Dakar)

This perceived lack of political will, in turn, was expressed at the meso-level as lack of information forthcoming from the Telemedicine National Steering Committee.

Relatedly, telemedicine was considered to less prioritized by government than universal health coverage, maternal health, infant health, malaria, HIV, tuberculosis and Ebola.

"No! Telemedicine is not a political priority. Currently, the political priority is universal health coverage. It is access to healthcare."

(Male specialist physician working in a district health centre outside Dakar)

Given how telemedicine can function to increase healthcare access, there is an evident disconnect between development of telemedicine in Senegal, and how it can meet the other political health priorities.

Many of the physicians participating in this study also noted that there is no national telemedicine strategy in Senegal.

"Now, there is no national telemedicine strategy. In any case, I am not aware. I don’t have any information. In my view, we must first set up this strategy."

(Male specialist physician working in a public hospital in Dakar)

For them, the lack of national strategy corresponds to the lack of directives on telemedicine. They do not see the necessity to use telemedicine because they are evaluated according to directives received from health authorities.

#### Financial factors

Financial factors are critical for the effective uptake of telemedicine. Telemedicine itself is costly and if there is little dedicated funding to sustain its use it is challenging to scale up. There are also costs associated with the time required by different health workers to utilize telemedicine.

Participants expressed concern that the use of telemedicine involves substantial investment, operating, training and maintenance costs. They believed that these costs could be exorbitant and become an obstacle to the development of the technology and its use. Concerning training costs alone, one of the telemedicine projects managers said:

"We have to organize a lot of training workshops, and these workshops require a lot of resources because we have to convene not only physicians from regions but also from health facilities to exchange with them. These workshops require a lot of money. They are organized in hotels. Sometimes, they last one day. Sometimes, they last several days."

(Male telemedicine projects manager working in Dakar)

These financial problems could be addressed somewhat by acting on legal factors such as revising existing laws or regulations, and lowering taxes related to telemedicine equipment, electricity, and the internet. This assumes that telemedicine becomes a political priority for the Senegalese government.

Health committees, local communities, hospitals, government and development partners are the usual sources of funding in the public health sector. Participants commented that health committees, local communities, and hospitals will not be able to finance telemedicine on their own due to existing financial difficulties:

"Hospital cannot finance. It has other problems. It is not able to finance even medical equipment. Even tensiometer, it is not able to pay."

(Female specialist physician working in a public hospital outside Dakar)

A small number of the interviewed physicians (n = 4) believed that they should be remunerated for overtime and private telemedicine practice. According to these physicians, the non-resolution of this issue can have a negative impact on their motivation to use telemedicine and consequently on the success of that technology.

"From my understanding, telemedicine will be part of our daily activities. In this case, we can register it in our schedule, but if one has to work overtime, he must be paid for that."

(Male specialist physician working in a public hospital in Dakar)

#### Socio-economic and cultural factors

Finally, the study identified four socioeconomic and cultural factors that can influence the use of telemedicine in Senegal. Our participants, for example, express how religious and socio-cultural beliefs can have a negative impact on the success of telemedicine by influencing patient decisions to not use the technology.

"There are some religious families that don’t use modern medicine. They use traditional medicine. There are also some families in which women and girls don’t see male physicians. These socio-cultural and religious beliefs are here and can hinder telemedicine use."

(Male general practitioner working in a district health centre outside Dakar)

Our results also identified strikes in health facilities as having an impact on the availability and use of telemedicine.

"When there are strikes, everybody leaves, and everything gets stuck. To make things, including telemedicine, work permanently, it is necessary to have stability in institutions. If there are ruptures because there are some imponderables such as strikes, this is a problem."

(Male specialist physician working in a public hospital in Dakar)

Broader forms of social conflict can also create a climate of stress that prevents providers from doing their work and therefore using telemedicine.

"There is also the conflict … that creates insecurity. These things can prevent us from working and using telemedicine. In 2011, we were invaded by rebels in the centre. Then, we were scared, and we could not go to work. We stayed, at least, one week at home. The staff did not go to work."

(Male general practitioner working in a district health centre outside Dakar)

Physicians also commented that many of their patients would be unable to pay for telemedicine services if these costs were too high. For them, the low purchasing power of rural people does not allow them to pay for costly telemedicine services.

"Here, people are poor. They don’t have money, and we know that they don’t have insurance to cover their costs. They have to pay everything and from their pockets."

(Male general practitioner working in a district health centre outside Dakar)

Rural poverty can have a negative impact on the sustainability of telemedicine services simply because there are user charges for health services in Senegal and most of the patients pay these charges directly from their pockets, unless they are covered by health insurance.

## Discussion

To examine the individual and contextual factors that determine the use of telemedicine in Senegal, this study used a micro, meso and macro framework adapted from the social-ecological framework of Dahlgren & Whitehead [41]. To the best of our knowledge, this study is the first to use this framework in examining the individual and contextual factors that determine the use of telemedicine in Senegal. Our findings broadly affirm those of other studies, including those that were undertaken primarily in high-income countries. First, we found that most of the physicians we interviewed who were working in public hospitals and district health centres were likely to use telemedicine in their professional activities. These results are consistent with the findings of other studies that the attitude of health professionals was more positive than negative toward telemedicine [42]. But acting on physicians’ intention required dealing with several disabling factors at the meso- and macro-level. Consistent with other research, many of the meso-level factors concerned a lack of technical capacity [36, 43] as well as a need for ethical guidance in the use of telemedicine [44]. Training in both was seen to be largely lacking in the medical curriculum.

An important macro-level barrier was the perceived failure of the Telemedicine National Steering Committee, which figured into the lack of political interest or prioritisation of telemedicine development, as well as an inability to coordinate across the many small-scale and overlapping telemedicine start-ups. The absence of a legal framework offering protections to physicians using the technology was considered another important barrier. Perhaps the greatest impediment to any scale-up of telemedicine in Senegal, however, are the significant investment, operating, maintenance and training costs, frequently mentioned by our interviewees and constantly referenced in the literature [22, 27]. The lack of compensation for physicians using telemedicine is known as another important barrier to its use [29], although was only a minority concern for our participants, which may be due to their employment within the public, and not the private, sector. Outside of publicly provided access, rural and remote populations rarely have sufficient income to pay out-of-pocket fees related to telemedicine [36].

These various contextual factors may interact with each other in interdependent ways, amplifying the barriers to telemedicine use. For example, a Senegalese physician who is interested in using telemedicine needs, at least, telemedicine equipment, electricity, internet connection, and training. The availability of these technical elements and the performance of that training are influenced by the availability of financial resources. This financial factor is recognized as one of the most important determinants of the success of telemedicine [23, 44]. It is, in turn, influenced by the political factors such as considering telemedicine as a political priority, translating political will into concrete action or having telemedicine policy and dedicated funding. It is well known that political factors such as the lack of policy can prevent the use of telemedicine [45].

This illustration shows that the use of telemedicine depends on meso factors, such as technical factors, which depend, in turn, on macro factors such as financial factors. It also shows that macro factors such as political and financial factors could interact with each other. These lead us to conclude that individual factors or micro-level factors (physician intentions) are influenced by a cascade of both meso- and macro-level factors. The results of this study, while largely descriptive, should prove useful for health system actors in developing interventions to improve the sustainable development of telemedicine in Senegal. Our study provides them with a more detailed idea on where actions should start if they want an impact on the bigger number of factors, for example, that acting on political factors can positively impact financial factors which can have a positive impact on the technical factors. Although these findings are limited by virtue of representing a single case, and by omitting for logistical reasons private practice physicians in the sampling design, they are sufficiently similar to other study results from high-income countries to represent potentially transferable knowledge for other resource-constrained settings besides Senegal.

## Conclusions

This study provides one of the most comprehensive insights on the individual and contextual factors that influence the use of telemedicine in resource-constrained settings by identifying and assessing the micro (physicians’ intention), meso (technical, organizational and ethical) and macro (financial, political, legal and socioeconomic) factors that determine the use of that technology in Senegal. Knowing these individual and contextual factors can assist actors in the health sector in their support for the development of telemedicine in Senegal. In turn, this can improve the recruitment and retention of physicians in underserved areas, which in time is likely to improve Senegal’s overall population health and particularly that of populations living in currently underserved regions.

## Supporting information

S1 File The quantitative questionnaire. The administrative questionnaire administered to the physicians working in public hospitals and district health centres to study their intention.
(PDF)

S2 File Interview guide used in the qualitative study involving individual interview. This interview guide was used to determine the meso (technical, organizational and ethical) and macro (financial, political, legal and socioeconomic) levels factors.
(PDF)

S3 File Data supporting the results related to the intention of the physicians working in public hospitals in Senegal. (PDF)

S4 File Data supporting the results related to the intention of the physicians working in district health centres in Senegal. (PDF)

S5 File Data supporting the socio-demographic and professional characteristics of the physicians working in public hospitals involved in the qualitative study (meso and macro-factors). (PDF)

S6 File Data supporting the socio-demographic and professional characteristics of the physicians working in district health centres involved in the qualitative study (meso and macro-factors). (PDF)

S7 File Data supporting the socio-demographic and professional characteristics of the telemedicine project managers involved in the qualitative study (meso and macro-factors). (PDF)
