# Supplementary material for: The individual and contextual determinants of the use of telemedicine: A descriptive study of the perceptions of Senegal's physicians and telemedicine projects managers
Source: PLoS One. 2017 Jul 21;12(7):e0181070. doi: 10.1371/journal.pone.0181070 (PMC5521789; doi:10.1371/journal.pone.0181070)
Supplement: S1 File — The administrative questionnaire administered to the physicians working in public hospitals and district health centres to study their intention. (PDF) [file pone.0181070.s001.pdf]

Questionnaire used to study the intention of the physicians working in public hospitals and district health centres to use telemedicine in their professional activities

Ce questionnaire est destiné à l'étude de l'intention des médecins des hôpitaux et des centres de santé de district du Sénégal d'utiliser la télémédecine dans leurs activités professionnelles. Nous vous prions de bien vouloir le remplir et nous le retourner, dans les deux semaines qui suivent sa réception, à l'adresse bly022@uottawa.ca ou apholyca@hotmail.com ou à la direction régionale de la santé. La confidentialité et l'anonymat de vos informations seront rigoureusement respectés.

- **La télémédecine est définie comme l'exercice de la médecine à distance. Elle implique l'utilisation des technologies de l'information et de la communication (ordinateur, téléphone, tablette, fax et autres) et englobe le diagnostic, le traitement, le suivi et la formation.**
- **Par activités professionnelles, nous entendons les activités de diagnostic, de traitement, de suivi et de formation qui font partie de vos devoirs de médecin des hôpitaux.**

1. N° du questionnaire : /___/___/ *(réservez à l'équipe de recherche)*

Pour répondre aux questions, inscrire la bonne réponse ou mettre une croix dans les espaces réservés à cet effet.

2. Date of administration of the questionnaire : /____/____/20___/
3. Phone Number : /__________________________________/
4. Email : /__________________________________/
5. Medical Region : /__________________________________/
6. Public hospital or district health centre :
/____/
7. Department : /____/
8. Birthday : /____/____/______/
9. Sex : Homme : /__/Femme : /__/
10. Spéciality : /____/
11. Number of years in the current hospital or district health centre: /_____/
12. Number of years in medical practice : /_____/
13. Professional Status : Civil servant /__/other /__/
If other, precise : /___________________________________/
14. I intent to use telemedicine in my professional activities during the next 12 months.

Highly unlikely	Quite unlikely	Slightly unlikely	Neither unlikely nor likely	Slightly likely	Quite likely	Highly likely
