# Supplementary material for: The individual and contextual determinants of the use of telemedicine: A descriptive study of the perceptions of Senegal's physicians and telemedicine projects managers
Source: PLoS One. 2017 Jul 21;12(7):e0181070. doi: 10.1371/journal.pone.0181070 (PMC5521789; doi:10.1371/journal.pone.0181070)
Supplement: S2 File — This interview guide was used to determine the meso (technical, organizational and ethical) and macro (financial, political, legal and socioeconomic) levels factors. (PDF) [file pone.0181070.s002.pdf]

Table 1: Interview guide for the study of the contextual factors that influence the use of telemedicine

Contextual factors	Contextual factors	Questions
**Meso**	**Technical**	What are the technical factors that influence the use of telemedicine in Senegal?
	**Organizational**	What are the organizational factors that influence the use of telemedicine in Senegal?
	**Ethical**	What are the ethical factors that influence the use of telemedicine in Senegal?
**Macro**	**Financial**	What are the financial factors that influence the use of telemedicine in Senegal?
	**Political**	What are the political factors that influence the use of telemedicine in Senegal?
	**Legal**	What are the legal factors that influence the use of telemedicine in Senegal?
	**Socioeconomic**	What are the socioeconomic factors that influence the use of telemedicine in Senegal?
