# Supplementary material for: The individual and contextual determinants of the use of telemedicine: A descriptive study of the perceptions of Senegal's physicians and telemedicine projects managers
Source: PLoS One. 2017 Jul 21;12(7):e0181070. doi: 10.1371/journal.pone.0181070 (PMC5521789; doi:10.1371/journal.pone.0181070)
Supplement: S3 File — (PDF) [file pone.0181070.s003.pdf]

The physicians working in public hospitals involved in the study of intention

No	Code	Q2	Region	Age	Sex	Speciality	Intention
1	H001	26-01-2015	Dakar	15-10-1963	M	Specialist Physician	2
2	H002	26-01-2015	Dakar	13-09-1975	F	Specialist Physician	1
3	H003	26-01-2015	Dakar	31-12-1963	M	Specialist Physician	3
4	H004	29-01-2015	Dakar	26-03-1967	M	Specialist Physician	1
5	H005	29-01-2015	Dakar	17-08-1971	F	Specialist Physician	2
6	H006	29-01-2015	Dakar	15-02-1979	M	Specialist Physician	2
7	H007	30-01-2015	Dakar	20-11-1982	M	Specialist Physician	1
8	H008	30-01-2015	Dakar	15-08-1978	M	Specialist Physician	3
9	H009	30-01-2015	Dakar	14-09-1984	F	Specialist Physician	2
10	H010	30-01-2015	Dakar	08-04-1967	F	Specialist Physician	-3
11	H011	29-01-2015	Dakar	21-11-1974	M	Specialist Physician	1
12	H012	30-01-2015	Dakar	18-03-1978	M	Specialist Physician	0
13	H013	02-02-2015	Out of Dakar	05-05-1979	M	Specialist Physician	3
14	H014	02-02-2015	Out of Dakar	23-05-2015	F	Specialist Physician	-1
15	H015	02-02-2015	Out of Dakar	10-02-1969	M	Specialist Physician	2
16	H016	02-02-2015	Out of Dakar	06-08-2015	M	Specialist Physician	3
17	H017	02-02-2015	Out of Dakar	22-05-1964	F	Specialist Physician	-2
18	H018	02-02-2015	Out of Dakar	28-02-1978	M	Specialist Physician	3
19	H019	02-02-2015	Out of Dakar	07-03-1975	M	Specialist Physician	2
20	H020	03-02-2015	Dakar	13-01-1977	F	Specialist Physician	0
21	H021	03-02-2015	Dakar	22-12-1967	F	Specialist Physician	-3
22	H022	03-02-2015	Dakar	20-04-1983	M	Specialist Physician	2
23	H023	03-02-2015	Dakar	28-06-1968	F	Specialist Physician	2
24	H024	03-02-2015	Dakar	05-07-1978	M	Specialist Physician	2
25	H025	02-02-2015	Dakar	28-01-1972	M	Specialist Physician	3
26	H026	04-02-2015	Dakar	24-08-1976	M	Specialist Physician	1
27	H027	04-02-2015	Dakar	24-03-1976	F	Specialist Physician	3
28	H028	04-02-2015	Dakar	18-11-1967	M	Specialist Physician	2
29	H029	02-02-2015	Out of Dakar	09-02-2015	M	Specialist Physician	3
30	H030	09-02-2015	Dakar	15-02-2015	M	Specialist Physician	-2
31	H031	09-02-2015	Dakar	13-12-1972	F	Specialist Physician	-2
32	H032	09-02-2015	Dakar	01-02-1977	F	Specialist Physician	2
33	H033	06-02-2015	Dakar	14-11-1964	F	Specialist Physician	2
34	H034	06-02-2014	Dakar	01-11-1975	M	Specialist Physician	3
35	H035	09-02-2015	Dakar		F	Specialist Physician	3
36	H036	09-02-2015	Dakar	12-10-1968	M	Specialist Physician	2
37	H037	06-02-2015	Dakar	05-03-1982	M	Specialist Physician	-3
38	H038	09-02-2015	Dakar	15-08-1979	M	Specialist Physician	-3
39	H039	06-02-2015	Dakar	17-01-1968	M	Specialist Physician	2
40	H040	06-02-2015	Dakar	28-07-1982	M	General Practitioner	2
41	H041	09-02-2015	Dakar	04-01-1977	M	Specialist Physician	3
42	H042	10-02-2015	Dakar	25-06-1984	M	Specialist Physician	3
43	H043	10-02-2015	Dakar	10-10-1968	F	Specialist Physician	-3
44	H044	10-02-2015	Dakar	16-01-1985	M	General Practitioner	-3
45	H045	10-02-2015	Dakar	13-02-1985	F	General Practitioner	1
46	H046	10-02-2015	Dakar	02-01-1968	F	Specialist Physician	-3
47	H047	10-02-2015	Dakar	10-10-1983	M	Specialist Physician	3
48	H048	11-02-2015	Dakar	14-07-1958	F	Specialist Physician	0
49	H049	11-02-2015	Dakar	23-04-1964	F	Specialist Physician	0
50	H050	11-02-2015	Dakar	03-12-2015	M	Specialist Physician	3
51	H051	04-02-2014	Dakar	18-09-1973	F	Specialist Physician	2
52	H053	23-01-2015	Dakar	06-09-1970	M	Specialist Physician	1
53	H054	26-01-2015	Dakar	02-06-1988	M	Specialist Physician	-1
54	H055	09-02-2015	Dakar	04-05-1961	M	Specialist Physician	1
55	H056	11-02-2015	Dakar	16-10-1967	F	Specialist Physician	2
56	H057	28-01-2015	Dakar	15-02-1972	M	Specialist Physician	1
57	H058	10-02-2015	Dakar	21-12-1968	F	Specialist Physician	2
58	H059	15-01-2015	Dakar	02-09-1978	M	Specialist Physician	-3
59	H060	28-01-2015	Dakar	23-11-1957	M	Specialist Physician	2
60	H061	11-02-2015	Dakar	06-05-1977	F	Specialist Physician	-2
61	H062	06-02-2015	Dakar	01-01-1961	M	Specialist Physician	3
62	H063	04-02-2015	Dakar	17-05-1982	F	General Practitioner	3
63	H064	10-02-2015	Dakar	03-09-1970	F	Specialist Physician	2
64	H065	11-02-2015	Dakar	15-09-1971	M	Specialist Physician	0
65	H066	09-02-2015	Dakar	10-06-1958	M	Specialist Physician	3
66	H067	28-01-2015	Dakar	10-06-1979	F	Specialist Physician	2
67	H068	02-02-2015	Dakar	03-02-1968	M	Specialist Physician	3
68	H069	04-02-2015	Dakar	06-11-1960	M	Specialist Physician	-3
69	H070	03-02-2015	Dakar	12-02-1980	M	Specialist Physician	2
70	H071	28-01-2015	Dakar	25-12-1970	F	Specialist Physician	3
71	H072	30-01-2015	Dakar	20-11-1961	M	Specialist Physician	3
72	H073	02-02-2015	Dakar	02-04-1956	M	Specialist Physician	-1
73	H074	26-01-2015	Dakar	25-06-1977	M	Specialist Physician	0
74	H075	09-02-2015	Dakar	03-05-1975	M	Specialist Physician	2
75	H076	09-02-2015	Dakar	08-02-1972	M	Specialist Physician	0
76	H077	09-02-2015	Dakar	26-12-1967	M	Specialist Physician	0
77	H078	29-01-2015	Dakar	28-12-1969	M	Specialist Physician	-3
78	H079	05-01-2015	Dakar	03-12-1973	M	Specialist Physician	1
79	H080	29-01-2015	Dakar	03-08-1983	M	Specialist Physician	1
80	H081	03-02-2015	Dakar	09-09-1970	F	Specialist Physician	1
81	H082	03-02-2015	Dakar	28-12-1974	M	Specialist Physician	-3
82	H083	03-02-2015	Dakar	26-10-1987	M	Specialist Physician	1
83	H084	28-01-2015	Dakar	10-01-1984	M	Specialist Physician	2
84	H085	02-02-2015	Dakar	19-10-1983	F	Specialist Physician	1
85	H086	30-01-2015	Dakar		M	Specialist Physician	2
86	H087	10-02-2015	Dakar	07-05-1982	F	Specialist Physician	2
87	H088	05-02-2015	Dakar	04-08-1978	M	Specialist Physician	3
88	H089	10-02-2015	Dakar	28-11-1985	F	General Practitioner	-2
89	H090	04-02-2015	Dakar	01-01-1962	M	Specialist Physician	1
90	H091	12-02-2015	Dakar	06-10-1978	F	Specialist Physician	2
91	H092	11-02-2015	Dakar	04-09-1978	M	Specialist Physician	-3
92	H093	13-02-2015	Dakar	03-03-1971	M	Specialist Physician	-2
93	H094	12-02-2015	Dakar	13-09-1977	M	Specialist Physician	1
94	H095	12-02-2015	Dakar	10-06-1969	M	Specialist Physician	0
95	H096	13-02-2015	Dakar	05-05-1972	M	Specialist Physician	3
96	H097	12-02-2015	Dakar	06-02-1961	F	Specialist Physician	1
97	H098	10-02-2015	Dakar	15-06-1962	F	Specialist Physician	0
98	H099	12-02-2015	Dakar	25-02-1953	M	Specialist Physician	-3
99	H100	13-02-2015	Dakar	20-04-1961	M	Specialist Physician	1
100	H101	04-02-2015	Dakar	06-07-1978	M	General Practitioner	2
101	H102	28-01-2015	Dakar	28-01-70	F	Specialist Physician	1
102	H103	03-02-2015	Dakar	05-12-1979	M	Specialist Physician	3
103	H104	28-01-2015	Dakar	02-01-1980	F	Specialist Physician	1
104	H105	02-02-2015	Dakar	14-02-1969	M	Specialist Physician	2
105	H106	30-01-2015	Dakar	11-07-1977	F	Specialist Physician	2
106	H107	29-01-2015	Dakar	02-04-1982	M	Specialist Physician	2
107	H108	03-02-2015	Dakar	19-12-1977	M	Specialist Physician	1
108	H109	02-02-2015	Dakar	04-05-1976	M	Specialist Physician	2
109	H110	04-02-2015	Dakar	23-03-1980	M	Specialist Physician	1
110	H111	03-02-2015	Dakar	12-09-1976	M	Specialist Physician	2
111	H112	23-01-2015	Dakar	13-03-1960	F	Specialist Physician	2
112	H113	05-02-2015	Dakar	03-03-1977	M	General Practitioner	-3
113	H114	06-02-2015	Dakar	22-07-1975	M	Specialist Physician	3
114	H115	04-02-2015	Dakar	01-04-1969	M	Specialist Physician	-2
115	H116	09-02-2015	Dakar	17-11-1970	M	Specialist Physician	2
116	H117	13-02-2015	Dakar	14-06-1983	M	Specialist Physician	3
117	H118	20-02-2015	Out of Dakar	20-05-1970	F	General Practitioner	1
118	H119	14-02-2015	Dakar	30-07-1988	F	General Practitioner	2
119	H120	20-02-2015	Out of Dakar	02-06-1982	M	General Practitioner	3
120	H121	16-02-2015	Out of Dakar	16-03-1984	F	General Practitioner	1
121	H122	16-02-2015	Out of Dakar	17-09-1972	F	Specialist Physician	2
122	H123	17-02-2015	Out of Dakar	07-05-1968	F	Specialist Physician	1
123	H124	16-02-2015	Out of Dakar	21-09-1975	F	Specialist Physician	2
124	H125	18-02-2015	Out of Dakar	08-01-1985	F	General Practitioner	1
125	H126	18-02-2015	Out of Dakar	28-03-1984	M	General Practitioner	2
126	H127	18-02-2015	Out of Dakar	15-10-1976	M	Specialist Physician	-3
127	H128	18-02-2015	Out of Dakar	16-02-1983	F	General Practitioner	2
128	H129	19-02-2015	Out of Dakar	07-06-1981	M	Specialist Physician	1
129	H130	19-02-2015	Out of Dakar	01-01-1982	M	Specialist Physician	3
130	H131	19-02-2015	Out of Dakar	24-03-1982	M	General Practitioner	3
131	H132	19-02-2015	Out of Dakar	02-02-1968	M	Specialist Physician	2
132	H133	19-02-2015	Out of Dakar	21-09-1973	F	Specialist Physician	2
133	H134	19-02-2015	Out of Dakar	10-12-1966	M	Specialist Physician	-2
134	H135	19-02-2015	Out of Dakar	10-03-1937	M	General Practitioner	2
135	H136	20-02-2015	Out of Dakar	08-10-1984	M	Specialist Physician	3
136	H137	20-02-2015	Out of Dakar	02-04-1985	M	General Practitioner	3
137	H138	05-02-2015	Out of Dakar	10-04-1970	M	Specialist Physician	1
138	H139	18-02-2015	Out of Dakar	01-01-1962	M	Specialist Physician	-2
139	H140	04-03-2015	Out of Dakar	02-04-1980	F	Specialist Physician	-3
140	H141	11-02-2015	Dakar	05-07-1986	F	General Practitioner	1
141	H142	09-02-2015	Dakar		F	Specialist Physician	0
142	H143	18-02-2015	Dakar	01-01-1981	M	Specialist Physician	2
143	H144	16-02-2015	Dakar	24-02-1983	M	Specialist Physician	2
144	H145	23-02-2015	Dakar	18-06-1979	M	Specialist Physician	0
145	H146	23-02-2015	Dakar	04-06-1974	M	Specialist Physician	1
146	H147	20-02-2015	Dakar	07-08-1983	F	General Practitioner	0
147	H148	17-02-2015	Dakar	26-02-1982	M	Specialist Physician	2
148	H149	20-02-2015	Dakar	03-06-1974	M	General Practitioner	2
149	H150	10-02-2015	Dakar	18-08-1984	F	General Practitioner	0
150	H151	03-03-2015	Dakar	08-05-1972	M	Specialist Physician	-3
151	H152	19-02-2015	Dakar	11-11-1978	M	General Practitioner	1
152	H153	17-02-2015	Dakar	25-02-1975	M	Specialist Physician	2
153	H154	06-03-2015	Dakar	03-03-1979	M	General Practitioner	-2
154	H155	04-03-2015	Dakar	10-07-1980	M	Specialist Physician	-3
155	H156	04-03-2015	Dakar	23-06-1984	F	General Practitioner	2
156	H158	21-03-2014	Out of Dakar	08-11-1974	M	Specialist Physician	3
157	H159	21-03-2015	Out of Dakar	17-02-1977	M	General Practitioner	3
158	H160	21-03-2015	Out of Dakar	13-05-1981	M	General Practitioner	1
159	H1161	21-03-2015	Out of Dakar	14-06-1983	M	General Practitioner	1
160	H163	21-03-2015	Out of Dakar	01-01-1980	M	General Practitioner	3
161	H164	20-03-2015	Out of Dakar	22-12-1969	M	Specialist Physician	3
162	H165	20-03-2015	Out of Dakar	15-05-1962	M	Specialist Physician	3
163	H167	19-03-2015	Out of Dakar	09-01-1984	M	General Practitioner	2
164	H168	18-03-2015	Out of Dakar	04-01-1984	M	General Practitioner	3
165	H169	19-03-2015	Out of Dakar	01-01-1976	M	Specialist Physician	0
