# Supplementary material for: The individual and contextual determinants of the use of telemedicine: A descriptive study of the perceptions of Senegal's physicians and telemedicine projects managers
Source: PLoS One. 2017 Jul 21;12(7):e0181070. doi: 10.1371/journal.pone.0181070 (PMC5521789; doi:10.1371/journal.pone.0181070)
Supplement: S5 File — (PDF) [file pone.0181070.s005.pdf]

The physicians working in public hospitals involved in the study of Contextual factors						
**N^{O}**	**Code D**	**Date**	**Age**	**Sex**	**Speciality**	**Region**
1	DS6	11-03-2014	38	M	Specialist Physician	Dakar
2	DS3	27-02-2014	53	M	Specialist Physician	Dakar
3	DS4	05-03-2014	29	F	Specialist Physician	Out of Dakar
4	DS1	28-02-2014	34	M	Specialist Physician	Dakar
5	DS5	07-03-2014	45	M	Specialist Physician	Out of Dakar
6	DS2	28-02-2014	46	M	Specialist Physician	Dakar
7	DS7	07-03-2014	46	M	Specialist Physician	Out of Dakar
8	DS8	07-03-2014	44	M	Specialist Physician	Out of Dakar
9	DS9	12-03-2014	44	F	Specialist Physician	Dakar
10	DS10	12-03-2014	43	F	Specialist Physician	Dakar
11	DS11	12-03-2014	41	M	Specialist Physician	Dakar
12	DS12	11-03-2014	52	M	Specialist Physician	Dakar
13	20114	11-04-2014	47	M	Specialist Physician	Dakar
14	20116	12-04-2014	37	M	Specialist Physician	Out of Dakar
15	20118	15-04-2014	50	M	Specialist Physician	Dakar
16	20120	15-04-2014	55	M	Specialist Physician	Dakar
17	20122	15-04-2014	44	M	Specialist Physician	Dakar
18	20124	16-04-2014	35	F	Specialist Physician	Dakar
19	20126	16-04-2014	51	M	Specialist Physician	Dakar
20	20128	16-04-2014	46	F	Specialist Physician	Dakar
21	20130	17-04-2014	52	M	Specialist Physician	Dakar
22	20132	17-04-2014	46	M	Specialist Physician	Dakar
23	20135	18-04-2014	54	M	Specialist Physician	Out of Dakar
24	20137	21-04-2014	32	M	Specialist Physician	Dakar
25	20139	22-04-2014	30	M	Specialist Physician	Dakar
26	20141	23-04-2014	61	M	Specialist Physician	Dakar
27	20145	25-04-2014	36	F	Specialist Physician	Dakar
28	20149	27-04-2014	51	M	Specialist Physician	Dakar
29	20151	29-04-2014	43	M	Specialist Physician	Dakar
30	20153	29-04-2014	40	M	Specialist Physician	Dakar
