# Supplementary material for: The individual and contextual determinants of the use of telemedicine: A descriptive study of the perceptions of Senegal's physicians and telemedicine projects managers
Source: PLoS One. 2017 Jul 21;12(7):e0181070. doi: 10.1371/journal.pone.0181070 (PMC5521789; doi:10.1371/journal.pone.0181070)
Supplement: S6 File — (PDF) [file pone.0181070.s006.pdf]

The physicians working in district health centres involved in the study of contextual factors

N[O]	Code D	Date	Region	Age	Sex	Speciality
1	DG1	3/4/2014	Out of Dakar	47	M	Specialist Physician
2	DG10	03-03-2014	Out of Dakar	39	M	General Practitioner
3	CG11	3/8/2014	Out of Dakar	42	M	Specialist Physician
4	DG13	04-03-2014	Out of Dakar	53	F	Specialist Physician
5	DG14	06-03-2014	Out of Dakar	39	M	Specialist Physician
6	DG15	28-02-2014	Out of Dakar	33	M	Specialist Physician
7	DG17	04-03-2014	Out of Dakar	42	M	Specialist Physician
8	DG18	03-03-2014	Out of Dakar	34	M	General Practitioner
9	DG19	03-03-2014	Out of Dakar	37	M	Specialist Physician
10	DG2	05-03-2014	Out of Dakar	36	M	Specialist Physician
11	DG20	03-03-2014	Out of Dakar	37	M	Specialist Physician
12	DG21	04-03-2014	Dakar	35	M	General Practitioner
13	DG24	03-03-2014	Out of Dakar	47	M	Specialist Physician
14	DG25	03-03-2014	Out of Dakar	35	M	General Practitioner
15	DG26	12-03-2014	Out of Dakar	37	M	Specialist Physician
16	DG27	28-02-2014	Out of Dakar	35	M	Specialist Physician
17	CG24	04-03-2014	Out of Dakar	40	M	Specialist Physician
18	DG28	05-03-2014	Out of Dakar	35	M	Specialist Physician
19	DG29	04-03-2014	Out of Dakar	37	M	Specialist Physician
20	DG31	05-03-2014	Out of Dakar	37	M	0
21	20104	28-03-2014	Out of Dakar	46	M	General Practitioner
22	20155	03-05-2014	Out of Dakar	47	M	Specialist Physician
23	20157	05-05-2014	Out of Dakar	39	M	General Practitioner
24	CG24	03-03-2014	Out of Dakar	34	M	General Practitioner
25	DG4	03-03-2014	Out of Dakar	34	M	Specialist Physician
26	DG5	05-03-2014	Out of Dakar	33	M	General Practitioner
27	DG6	03-03-2014	Dakar	44	F	Specialist Physician
28	DG7	06-03-2014	Out of Dakar	41	M	Specialist Physician
29	DG8	04-03-2014	Out of Dakar	44	M	General Practitioner
30	DG9	05-03-2014	Out of Dakar	44	M	General Practitioner
31	CG11	02-02-2014	Out of Dakar	33	M	Specialist Physician
32	DG30	11-03-2014	Out of Dakar	43	M	General Practitioner
33	CG30	04-03-2014	Out of Dakar	40	M	Specialist Physician
34	20108	29-03-2014	Out of Dakar	48	M	Specialist Physician
35	20106	29-03-2014	Out of Dakar	39	M	General Practitioner
36	20102	28-03-2014	Out of Dakar	39	M	General Practitioner
