# Supplementary material for: The individual and contextual determinants of the use of telemedicine: A descriptive study of the perceptions of Senegal's physicians and telemedicine projects managers
Source: PLoS One. 2017 Jul 21;12(7):e0181070. doi: 10.1371/journal.pone.0181070 (PMC5521789; doi:10.1371/journal.pone.0181070)
Supplement: S7 File — (PDF) [file pone.0181070.s007.pdf]

| The telemedicine project managers involved in the study of contextual factors | | | | | | |
|---|---|---|---|---|---|---|
| **N$^{O}$** | **Code D** | **Date** | **Region** | **Age** | **Sex** | **Speciality** |
| 1 | 20147 | 4/25/2014 | Dakar | 56 | M | Specialist Physician |
| 2 | 20146 | 4/25/2014 | Dakar | 63 | M | Specialist Physician |
| 3 | 20143 | 4/23/2014 | Dakar | 35 | M | ICT Specialist |
| 4 | 20158 | 5/9/2014 | Dakar | 56 | M | Specialist Physician |
| 5 | 20159 | 5/9/2014 | Dakar | 60 | M | Specialist Physician |
| 6 | TPM1 | 5/17/2014 | Out of Dakar | 53 | M | Specialist in Education Sciences (Option: didactic of sciences and elearning) |
| 7 | 20161 | 5/16/2014 | Out of Dakar | 50 | M | Specialist Physician |
| 8 | 20164 | 5/28/2014 | Dakar | 53 | M | Specialist Physician |
| 9 | TPM2 | 5/26/2014 | Dakar | 47 | M | Specialist Physician |
| 10 | TPM3 | 5/14/2014 | Dakar | 29 | M | Health Business Developer |
