# Supplementary material for: The individual and contextual determinants of the use of telemedicine: A descriptive study of the perceptions of Senegal's physicians and telemedicine projects managers
Source: PLoS One. 2017 Jul 21;12(7):e0181070. doi: 10.1371/journal.pone.0181070 (PMC5521789; doi:10.1371/journal.pone.0181070)
Supplement: S4 File — (PDF) [file pone.0181070.s004.pdf]

The physicians working in district health centres involved in the study of intention							
No	Code	Date	Region	Age	Sex	Speciality	Intention
1	D001	25-02-2015	Dakar	04-10-1976	F	Specialist Physician	2
2	D002	25-02-2015	Dakar	26-01-1972	M	General Practitioner	1
3	D003	25-02-2015	Dakar	18-03-1959	M	Specialist Physician	1
4	D004	25-02-2015	Dakar	20-01-1967	F	General Practitioner	1
5	D005	25-02-2015	Out of Dakar	23-01-1975	M	General Practitioner	3
6	D006	24-02-2015	Dakar	01-01-1978	M	Specialist Physician	2
7	D007	24-02-2015	Out of Dakar	19-01-1963	M	General Practitioner	-1
8	D008	23-02-2015	Out of Dakar	12-01-1968	M	Specialist Physician	3
9	D009	24-01-2015	Out of Dakar	24-02-1981	F	General Practitioner	3
10	D010	21-02-2015	Out of Dakar	04-02-1976	M	Specialist Physician	2
11	D011	20-02-2015	Out of Dakar	07-05-1976	M	Specialist Physician	1
12	D012	20-02-2015	Out of Dakar	05-01-1980	M	Specialist Physician	1
13	D013	20-02-2015	Out of Dakar	10-04-1978	F	General Practitioner	1
14	D014	20-02-2015	Out of Dakar	28-12-1982	M	General Practitioner	1
15	D015	20-02-2015	Out of Dakar	26-02-1975	M	General Practitioner	2
16	D016	18-02-2015	Out of Dakar	10-01-1983	M	General Practitioner	3
17	D017	18-02-2015	Out of Dakar	11-11-1962	F	Specialist Physician	-3
18	D018	19-02-2015	Out of Dakar	31-12-1977	M	Specialist Physician	1
19	D019	19-02-2015	Out of Dakar	30-10-1969	M	Specialist Physician	-3
20	D020	16-02-2015	Out of Dakar	02-04-1975	M	Specialist Physician	2
21	D021	14-02-2015	Out of Dakar	03-01-1976	M	Specialist Physician	-3
22	D022	08-02-2015	Out of Dakar	07-06-1971	M	General Practitioner	1
23	D023	19-02-2015	Out of Dakar	09-09-1983	M	General Practitioner	1
24	D024	20-02-2015	Out of Dakar	29-09-1981	M	Specialist Physician	3
25	D025	16-02-2015	Out of Dakar	07-10-1981	M	General Practitioner	2
26	D026	18-02-2015	Out of Dakar	28-11-1981	M	General Practitioner	3
27	D027	18-02-2015	Out of Dakar	10-07-1961	M	Specialist Physician	1
28	D028	18-02-2015	Out of Dakar	10-05-1960	F	Specialist Physician	1
29	D029	18-02-2015	Out of Dakar	11-10-1978	F	Specialist Physician	2
30	D030	18-02-2015	Out of Dakar	28-03-1976	M	Specialist Physician	1
31	D031	18-02-2015	Out of Dakar	14-01-1981	F	General Practitioner	2
32	D032	17-02-2015	Out of Dakar	11-05-1980	M	Specialist Physician	3
33	D033	18-02-2015	Out of Dakar	06-05-1980	M	General Practitioner	3
34	D034	17-02-2015	Out of Dakar	02-01-1980	M	General Practitioner	3
35	D035	17-02-2015	Out of Dakar	29-01-1975	M	Specialist Physician	1
36	D036	17-02-2015	Out of Dakar	05-01-1970	M	General Practitioner	1
37	D037	16-02-2015	Out of Dakar	02-12-1963	M	General Practitioner	1
38	D038	17-02-2015	Out of Dakar	26-11-1966	M	Specialist Physician	1
39	D039	19-02-2015	Out of Dakar	01-04-1978	M	Specialist Physician	1
40	D040	19-02-2015	Out of Dakar	30-12-1967	M	Specialist Physician	2
41	D041	17-02-2015	Out of Dakar	01-08-1976	M	Specialist Physician	-3
42	D042	21-02-2015	Out of Dakar	03-01-1984	M	General Practitioner	1
43	D043	17-02-2015	Out of Dakar	18-07-1979	F	Specialist Physician	1
44	D044	17-02-2015	Out of Dakar	01-04-1979	F	General Practitioner	-2
45	D045	16-02-2015	Out of Dakar	25-02-1983	M	General Practitioner	2
46	D046	12-02-2015	Out of Dakar	13-03-1980	M	General Practitioner	3
47	D047	16-02-2015	Out of Dakar	31-03-1973	M	Specialist Physician	-3
48	D048	17-02-2015	Out of Dakar	02-06-1980	M	Specialist Physician	1
49	D049	17-02-2015	Out of Dakar	01-03-1980	M	General Practitioner	2
50	D050	17-02-2015	Out of Dakar	09-12-1976	M	General Practitioner	0
51	D051	17-02-2015	Out of Dakar	28-07-1979	M	General Practitioner	-2
52	D052	26-02-2015	Dakar	17-03-1965	F	Specialist Physician	3
53	D053	26-02-2015	Dakar	04-02-1986	F	Specialist Physician	1
54	D054	26-02-2015	Dakar	18-06-1978	F	Specialist Physician	1
55	D055	26-02-2015	Dakar	15-11-1965	F	Specialist Physician	2
56	D056	26-02-2015	Dakar	14-11-1977	F	Specialist Physician	3
57	D057	26-02-2015	Dakar	05-09-1974	M	Specialist Physician	3
58	D058	26-02-2015	Dakar	21-05-1966	F	General Practitioner	2
59	D059	23-02-2015	Dakar	16-01-1974	F	Specialist Physician	2
60	D060	26-02-2015	Dakar	01-01-1963	M	Specialist Physician	1
61	D061	27-02-2015	Dakar	15-05-1969	F	Specialist Physician	3
62	D062	26-02-2015	Dakar	19-11-1975	F	Specialist Physician	1
63	D063	26-02-2015	Dakar	20-03-1979	M	General Practitioner	1
64	D064	25-02-2015	Dakar	17-11-1986	F	Specialist Physician	1
65	D065	26-02-2015	Dakar	11-12-1976	M	Specialist Physician	2
66	D066	26-02-2015	Dakar	14-11-1958	M	Specialist Physician	1
67	D067	27-02-2015	Dakar	18-12-1962	M	Specialist Physician	1
68	D068	02-03-2015	Dakar	10-11-1975	F	Specialist Physician	3
69	D069	02-03-2015	Dakar	21-07-1974	M	General Practitioner	2
70	D070	02-03-2015	Dakar	18-08-1966	F	General Practitioner	1
71	D071	02-03-2015	Dakar	09-11-1983	M	General Practitioner	2
72	D072	27-02-2015	Dakar	27-09-1979	F	Specialist Physician	2
73	D073	03-03-2015	Out of Dakar	09-01-1968	M	Specialist Physician	3
74	D074	04-03-2015	Out of Dakar	31-01-1975	F	Specialist Physician	-2
75	D075	04-03-2015	Out of Dakar	02-06-1975	M	Specialist Physician	-2
76	D076	03-03-2015	Out of Dakar	19-10-1978	F	General Practitioner	1
77	D077	04-03-2015	Out of Dakar	09-07-1973	F	General Practitioner	-2
78	D078	03-03-2015	Out of Dakar	04-09-1979	F	General Practitioner	-1
79	D079	03-03-2015	Out of Dakar	08-10-1974	M	General Practitioner	2
80	D080	03-03-2015	Out of Dakar	21-01-1978	M	Specialist Physician	1
81	D081	09-03-2015	Dakar	12-04-1973	F	Specialist Physician	-3
82	D082	09-03-2015	Dakar	03-12-1959	M	Specialist Physician	2
83	D083	09-03-2015	Dakar	03-01-1982	F	General Practitioner	3
84	D084	09-03-2015	Dakar	04-09-1970	F	Specialist Physician	-2
85	D085	09-03-2015	Dakar	21-08-1981	M	Trainee Physician	3
86	D086	07-02-2015	Out of Dakar	14-06-1984	M	Trainee Physician	2
87	D087	08-02-2015	Out of Dakar	07-12-1981	M	General Practitioner	2
88	D088	09-03-2015	Dakar	05-09-1968	F	Specialist Physician	1
89	D089	10-03-2015	Dakar	13-01-1981	M	Specialist Physician	1
90	D090	10-03-2015	Dakar	11-09-1982	F	Specialist Physician	2
91	D091	10-03-2015	Dakar	08-02-1988	F	General Practitioner	-2
92	D092	10-03-2015	Dakar	12-01-1969	M	Specialist Physician	3
93	D093	10-03-2015	Dakar	24-11-1973	F	Specialist Physician	2
94	D094	10-03-2015	Dakar	11-08-1979	M	Specialist Physician	3
95	D095	10-03-2015	Dakar	23-08-1978	F	Specialist Physician	1
96	D096	11-03-2015	Dakar	02-05-1985	F	General Practitioner	1
97	D097	10-03-2015	Dakar	13-08-1988	F	Specialist Physician	0
98	D098	03-03-2015	Dakar	12-02-1980	M	Specialist Physician	-2
99	D099	12-03-2015	Dakar	12-06-1968	M	Specialist Physician	3
100	D100	12-03-2015	Dakar	22-10-1970	F	Specialist Physician	3
101	D101	05-03-2015	Dakar	08-02-1961	M	Specialist Physician	-3
102	D102	13-03-2015	Dakar	29-12-1981	M	General Practitioner	1
103	D103	12-03-2015	Dakar	12-02-1977	F	General Practitioner	2
104	D104	13-03-2015	Dakar	24-03-1982	F	General Practitioner	1
105	D105	12-03-2015	Dakar	05-01-1979	F	General Practitioner	2
106	D106	13-03-2015	Dakar	04-06-1975	F	General Practitioner	2
107	D107	24-01-2015	Out of Dakar	05-01-1988	M	Trainee Physician	1
108	D108	05-02-2015	Out of Dakar	03-04-1984	M	Trainee Physician	2
109	D109	05-02-2015	Out of Dakar	03-09-1987	M	Trainee Physician	3
110	D110	21-02-2015	Out of Dakar	14-09-1987	F	Trainee Physician	1
111	D111	20-02-2015	Out of Dakar	21-06-1988	M	Trainee Physician	1
112	D112	20-02-2015	Out of Dakar	09-03-1986	F	Trainee Physician	2
113	D113	19-02-2015	Out of Dakar	16-01-1987	F	Trainee Physician	1
114	D114	19-02-2015	Out of Dakar	13-05-1987	F	Trainee Physician	3
115	D115	20-02-2015	Out of Dakar	03-10-1987	F	Trainee Physician	-3
116	D117	17-02-2015	Out of Dakar	25-04-1983	M	Trainee Physician	2
117	D118	18-02-2015	Dakar	03-12-1984	M	Trainee Physician	2
118	D119	21-03-2015	Out of Dakar	28-11-1987	F	General Practitioner	1
119	D120	20-03-2015	Out of Dakar	14-11-1968	F	Specialist Physician	3
120	D121	18-08-2015	Out of Dakar	12-07-1962	M	Specialist Physician	2
121	D122	20-03-2015	Out of Dakar	23-11-1986	F	General Practitioner	3
122	D123	20-03-2015	Out of Dakar	18-12-1963	M	General Practitioner	3
123	D124	20-03-2015	Out of Dakar	31-10-1966	M	Specialist Physician	-2
124	D125	19-03-2015	Out of Dakar	21-03-1973	M	Specialist Physician	1
125	D126	19-03-2015	Out of Dakar	10-07-1982	M	General Practitioner	-1
126	D127	19-03-2015	Out of Dakar	21-10-1970	M	General Practitioner	1
127	D128	19-03-2015	Out of Dakar	21-10-1966	M	Specialist Physician	2
128	D129	19-03-2015	Out of Dakar	23-05-1974	M	General Practitioner	3
129	D130	19-03-2015	Out of Dakar	04-12-1982	M	General Practitioner	3
130	D131	19-03-2015	Out of Dakar	05-03-1983	M	General Practitioner	-1
131	D132	19-03-2015	Out of Dakar	04-12-1986	M	General Practitioner	2
132	D133	19-03-2015	Out of Dakar	13-04-1982	M	General Practitioner	1
133	D134	19-03-2015	Out of Dakar	31-03-1970	M	Specialist Physician	3
134	D135	19-03-2015	Out of Dakar	12-12-1978	M	General Practitioner	2
135	D136	19-03-2015	Out of Dakar	15-09-1977	F	Specialist Physician	3
136	D137	19-03-2015	Out of Dakar	23-08-1983	M	General Practitioner	3
137	D138	18-03-2015	Out of Dakar	05-12-1984	M	General Practitioner	2
138	D139	18-03-2015	Out of Dakar	18-05-1962	M	Specialist Physician	-3
139	D140	16-03-2015	Out of Dakar	10-12-1983	M	General Practitioner	1
140	D141	21-03-2015	Out of Dakar	12-07-1976	M	Specialist Physician	3
141	D142	18-03-2015	Out of Dakar	31-01-1979	M	General Practitioner	3
142	D143	17-03-2015	Out of Dakar	27-03-1974	F	General Practitioner	-2
143	D144	17-03-2015	Out of Dakar	23-11-1971	F	Specialist Physician	3
144	D146	18-03-2015	Out of Dakar	17-06-1967	M	Specialist Physician	-1
145	D147	16-03-2015	Out of Dakar	15-11-1976	M	General Practitioner	1
146	D148	17-03-2015	Out of Dakar	27-01-1972	M	General Practitioner	2
147	D149	21-03-2015	Out of Dakar	08-01-1975	M	Specialist Physician	-3
148	D150	15-03-2015	Out of Dakar	04-05-1977	M	General Practitioner	2
149	D151	23-03-2015	Out of Dakar	01-01-1980	F	General Practitioner	1
150	D152	24-03-2015	Out of Dakar	27-05-1977	M	General Practitioner	1
151	D153	23-03-2015	Out of Dakar	17-09-1975	M	Specialist Physician	3
